# Effect of prior HoLEP procedure on multiparametric MRI accuracy in detection of prostate cancer

**DOI:** 10.1002/bco2.70105

**Published:** 2025-11-04

**Authors:** Austin Drysch, Kathryn E. Fink, Nicole Handa, Mitchell M. Huang, Sai Kumar, Yutai Li, Ridwan Alam, Amy E. Krambeck, Hiten D. Patel, Ashley E. Ross

**Affiliations:** ^1^ Feinberg School of Medicine, Department of Urology Northwestern University Chicago Illinois USA

**Keywords:** diagnostic accuracy, HoLEP, multiparametric MRI, PI‐RADS, prostate cancer

## Abstract

**Objectives:**

The objective of this study is to evaluate whether prior Holmium laser enucleation of the prostate (HoLEP) affects the diagnostic accuracy of multiparametric prostate MRI (mpMRI) with PI‐RADS scoring for detecting clinically significant prostate cancer (csPCa) on biopsy.

**Patients and Methods:**

We queried the Northwestern Electronic Data Warehouse for all patients who underwent mpMRI followed by prostate biopsy. Demographic information, mpMRI data including PI‐RADS score and biopsy data including Gleason grade (GG) were collected. Patients were stratified based on prior HoLEP and highest PI‐RADS score of index lesion on MRI. The outcome of interest was detection of csPCa (GG ≥ 2) on biopsy. Logistic regression was performed to assess the impact of prior HoLEP on the detection of csPCa at time of biopsy.

**Results:**

A total of 8937 patients met inclusion criteria, of which 97 patients (1.1%) had prior HoLEP. HoLEP specimen revealed benign pathology in 38 patients (39.2%), GG1 in 32 patients (33.0%), GG2 in 25 patients (25.8%) and GG3 in 2 patients (2.1%). Average time from HoLEP to mpMRI was 11.5 months. The post‐HoLEP group had lower prostate volumes (median 25.0 vs. 47.0 cc; *p* < 0.001) and PSA density (median 0.06 vs. 0.12 ng/ml^2^; *p* < 0.001). Rates of csPCa detection based on highest PI‐RADS score were comparable between control and HoLEP groups. Prior HoLEP did not significantly affect the detection of csPCa on multivariable analysis adjusting for age, race, PI‐RADS, family history of PCa, and PSA density (OR = 0.97; 95% CI: 0.60–1.57).

**Conclusion:**

PI‐RADS remains a reliable predictor of csPCa after HoLEP despite anatomic alterations. mpMRI should continue to guide biopsy and risk stratification in this population, though larger validation is warranted.

## 1 INTRODUCTION

Multiparametric prostate MRI (mpMRI) is recommended prior to prostate biopsy to optimize diagnostic accuracy and reduce unnecessary procedures.^1^, ^2^, ^3^, ^4^, ^5^ The Prostate Imaging Reporting and Data System (PI‐RADS) is an essential tool for characterizing lesions suspicious for clinically significant prostate cancer (csPCa) on mpMRI.^6^, ^7^ With the increasing use of mpMRI as a diagnostic tool, it is crucial to understand factors that might affect its accuracy.

Holmium laser enucleation of the prostate (HoLEP) is a minimally invasive procedure utilized in the treatment of bladder outlet obstruction secondary to benign prostatic hyperplasia (BPH).^8^ As HoLEP becomes increasingly prevalent due to its efficacy, safety profile and durability, understanding its impact on downstream diagnostic tools such as mpMRI is of growing clinical importance.^9^ Notably, incidental PCa is detected in up to 20% of patients undergoing HoLEP.^10^ While highly effective in relieving urinary symptoms, HoLEP, like other surgical treatments for BPH, results in structural changes to the prostate gland. These changes include gland deformity, residual adenoma, post‐surgical haemorrhages and potential chronic inflammatory changes, all of which can interfere with the interpretation of prostate mpMRI. These alterations may lower the accuracy of mpMRI by mimicking malignancy or obscuring true lesions, potentially reducing the positive predictive value (PPV) of PI‐RADS.^11^, ^12^, ^13^, ^14^

The present study evaluates the diagnostic performance of PI‐RADS scoring in patients who previously underwent HoLEP, using a large, contemporary cohort. Specifically, we aim to determine whether prior HoLEP significantly alters the predictive accuracy of mpMRI for detecting csPCa on targeted and systematic biopsy. We hypothesize that, despite anatomic alterations from HoLEP, mpMRI interpreted using PI‐RADS criteria remains a reliable tool for risk stratification and detection of csPCa.

## 2 PATIENTS AND METHODS

### 2.1 Study design and population

>This retrospective cohort study was approved by the Northwestern University Institutional Review Board and conducted using data from the Northwestern Electronic Data Warehouse. Patients were included if they underwent prostate mpMRI followed by biopsy between January 2020 and July 2025. Inclusion criteria required a PI‐RADS score on mpMRI and biopsy results indicating Gleason Grade (GG) or no cancer. Patients were stratified into two groups based on their history of HoLEP: (1) no prior HoLEP and (2) prior HoLEP procedure. Exclusion criteria included insufficient mpMRI or biopsy data, history of other prostate surgeries or incomplete clinical records. All data were de‐identified prior to analysis.

### 2.2 mpMRI protocol and PI‐RADS scoring

All mpMRI examinations were performed on 3‐T MRI scanners using a standardized protocol based on the European Society of Urogenital Radiology (ESUR) guidelines.^15^ Imaging sequences included T2‐weighted (T2W), diffusion‐weighted imaging (DWI), apparent diffusion coefficient (ADC) maps, and dynamic contrast‐enhanced (DCE) imaging. Lesions were scored by experienced radiologists using PI‐RADS Version 2.1 criteria, and the highest PI‐RADS score for each patient was recorded.^6^

### 2.3 Biopsy technique

All patients underwent prostate biopsies including systematic and, where applicable, targeted cores. Systematic biopsies (SBx) were obtained using a sextant or extended sampling scheme. Targeted biopsies (TBx) of lesions were performed using a software‐based fusion of mpMRI and transrectal ultrasound (TRUS) images. Biopsies were performed by either transrectal or transperineal approach at the discretion of the treating urologist. Histopathological evaluation was performed by dedicated genitourinary pathologists to determine Gleason Grade (GG) and classify csPCa (GG ≥ 2).

### 2.4 Data collection

Demographic data (age, race and BMI), clinical variables (prostate‐specific antigen [PSA], prostate volume and PSA density [PSAD]), imaging results (PI‐RADS score) and biopsy outcomes (Gleason Grade) were collected. HoLEP‐related variables, including time since surgery, were also recorded to assess their influence on mpMRI accuracy and biopsy outcomes.

### 2.5 Study outcomes

The primary outcome was the detection rate of csPCa (GG ≥ 2) on biopsy following mpMRI between patients with naïve prostate and patients with a history of HoLEP procedure. Secondary outcomes included the predictive value of PI‐RADS scores in detecting csPCa and the impact of HoLEP on mpMRI accuracy.

### 2.6 Statistical analysis

Descriptive statistics were used to summarize baseline characteristics and imaging findings between groups. Continuous variables were compared using the Wilcoxon rank‐sum test, while categorical variables were analysed using Fisher's exact test. Logistic regression models were employed to assess the relationship between HoLEP and csPCa detection, controlling for confounding factors such as age, race, PI‐RADS score, PSAD and family history of PCa. Predicted probabilities of csPCa were modelled by PI‐RADS score and HoLEP status. Statistical significance was set at *p* < 0.05. Statistical analyses were performed using R (Version 4.4.1).

## 3 RESULTS

A total of 8937 patients underwent prostate mpMRI followed by biopsy and met inclusion criteria. Among these, 97 patients (1.1%) had a history of prior HoLEP. Compared to patients without prior HoLEP, those in the HoLEP group were older (median 70.0 vs. 68.0; *p* = 0.007) and had lower prostate volumes (median 25.0 vs. 47.0 cc; *p* < 0.001) and PSAD (median 0.06 vs. 0.12 ng/ml^2^; *p* < 0.001) (Table 1). Median time from HoLEP to mpMRI was 11.5 months. Demographics including race, ethnicity, BMI and family history of prostate cancer were similar between groups (Table 1).

**TABLE 1:** Patient characteristics by prior HoLEP.

	No prior HoLEP	Prior HoLEP	*P* value^b^
Characteristic	*N* = 8840^a^	*N* = 97^a^	
Age	68.00 (63.00, 74.00)	70.00 (66.00, 76.00)	0.007
BMI	27.83 (25.22, 30.99)	27.33 (24.36, 30.25)	0.12
MRI prostate volume	47.00 (34.00, 67.00)	25.00 (18.50, 32.80)	<0.001
PSA density	0.12 (0.08, 0.19)	0.06 (0.03, 0.11)	<0.001
PI‐RADS			<0.001
1–2	1407 (16%)	18 (19%)	
3	1785 (20%)	19 (20%)	
4	3641 (41%)	54 (56%)	
5	2007 (23%)	6 (6.2%)	
Biopsy Gleason grade			0.036
No cancer	2945 (33%)	43 (44%)	
1	1326 (15%)	20 (21%)	
2	2198 (25%)	14 (14%)	
3	1303 (15%)	12 (12%)	
4	548 (6.2%)	3 (3.1%)	
5	520 (5.9%)	5 (5.2%)	
Any prostate cancer	5895 (67%)	54 (56%)	0.022
csPCa	4569 (52%)	34 (35%)	0.001

^a^ Median (Q1, Q3); *n* (%).

^b^ Wilcoxon rank sum test; Fisher's exact test; Pearson's Chi‐squared test.

Histopathologic evaluation of HoLEP specimens revealed GG1 in 32 patients (33.0%), GG2 in 25 patients (25.8%), and GG3 in 2 patients (2.1%), indicating that 59 patients (60.8%) included in the HoLEP cohort had an incidental diagnosis of PCa discovered after HoLEP but prior to mpMRI. The remaining 38 patients (39.2%) had benign HoLEP pathology. Among all patients, rates of both PCa (55.7% vs. 66.7%; *p* = 0.022) and csPCa (GG ≥ 2; 35.1% vs. 51.7%; *p* = 0.001) were significantly lower in the HoLEP group compared to the control group (Table 1).

**FIGURE 1:**
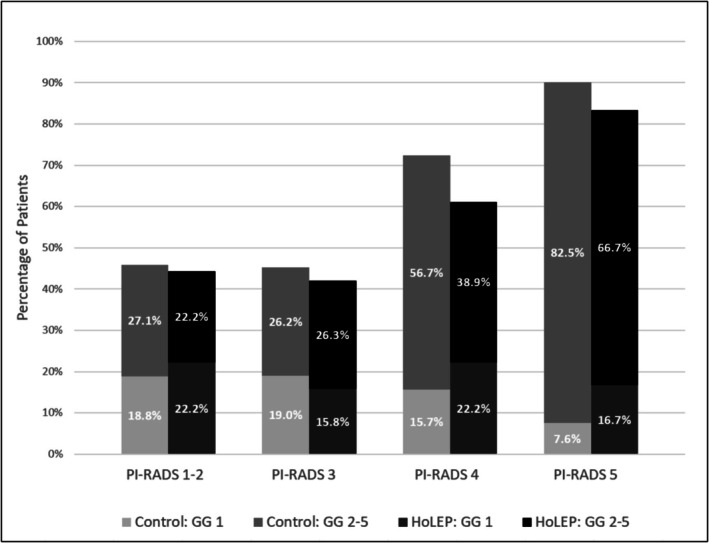
Highest PI‐RADS versus Gleason grade. Bar graph comparing csPCa detection rates post‐mpMRI biopsy across PI‐RADS categories (1–5) for patients with and without a history of HoLEP. In both groups, csPCa detection increased with higher PI‐RADS scores. While absolute detection rates were lower in the HoLEP group for most PI‐RADS categories, the overall pattern of increasing cancer risk with increasing PI‐RADS score was preserved.

PI‐RADS distribution also differed by HoLEP status. Only 6.2% of HoLEP patients had a PI‐RADS 5 lesion, compared to 22.7% of those without HoLEP (*p* < 0.001). Nonetheless, the predictive relationship between PI‐RADS score and csPCa detection was preserved in both groups. When stratified by PI‐RADS score, csPCa detection increased with higher PI‐RADS categories in both the HoLEP and non‐HoLEP groups. csPCa detection rates were 27.1% for PI‐RADS 1–2, 26.2% for PI‐RADS 3, 56.7% for PI‐RADS 4 and 82.5% for PI‐RADS 5 lesions in the control group. In the HoLEP group, corresponding csPCa detection rates were 22.2%, 26.3%, 38.9% and 66.7%, respectively (Figure 1).

On univariable logistic regression, prior HoLEP was associated with reduced odds of PCa detection (OR = 0.63; 95% CI: 0.42–0.94; *p* = 0.023), as well as csPCa detection (OR = 0.50; 95% CI: 0.33–0.77; *p* = 0.001). However, neither association was significant in a multivariable model adjusting for age, race, PI‐RADS score, family history of prostate cancer and log‐transformed PSAD (csPCa: OR = 0.97; 95% CI: 0.60–1.57; *p* = 0.90). Increasing age, higher PI‐RADS score, family history of prostate cancer and Black or African American race were associated with increased odds of csPCa in the multivariable model (Table 2).

**TABLE 2:** Multivariable logistic regression analysis by csPCa.

Characteristic	*N*	OR	95% CI	*P* value
Age	8454	1.032	1.026, 1.039	<0.001
Cohort	8454			
No Prior HoLEP	8360	—	—	
HoLEP Prior to mpMRI	94	0.966	0.595, 1.568	0.9
Race	8454			<0.001
White	6080	—	—	
Asian	231	0.723	0.527, 0.991	0.044
Black or African American	920	1.536	1.305, 1.808	<0.001
None of the above	533	0.930	0.757, 1.144	0.5
Patient unable/declined to respond	690	1.341	1.113, 1.616	0.002
PIRADS Recoded	8454			<0.001
1 or 2	1320	—	—	
3	1714	1.093	0.919, 1.301	0.3
4	3529	3.726	3.210, 4.326	<0.001
5	1891	9.697	8.081, 11.63	<0.001
Family history of prostate cancer	8454			
0	6643	—	—	
1	1811	1.390	1.231, 1.571	<0.001
logPSAD	8454	2.442	2.260, 2.638	<0.001

Abbreviations: CI, confidence interval; OR, odds ratio.

An interaction model evaluating whether the predictive value of PI‐RADS differed by HoLEP status did not reveal any significant effect modification, though the model was underpowered due to the small HoLEP sample size.

## 4 DISCUSSION

This study evaluated whether prior HoLEP impacts the diagnostic performance of mpMRI interpreted using PI‐RADS criteria for detecting csPCa. Despite anatomical changes resulting from HoLEP, our findings suggest that the predictive value of PI‐RADS for csPCa remains intact.

Patients with a history of HoLEP had significantly lower prostate volume, consistent with prior enucleation. As expected, overall PCa and csPCa detection was lower in the HoLEP group, likely due to PCa screening occurring prior to HoLEP. When stratified by PI‐RADS score, detection rates increased with higher PI‐RADS categories in both groups, supporting the retained discriminative utility of PI‐RADS scoring. Although fewer HoLEP patients had high‐risk lesions (only 6.2% had PI‐RADS 5 compared to 23% in the control group), PI‐RADS 5 had by far the highest odds of csPCa in both the HoLEP and control groups at 66.7% and 82.5%, respectively. Multivariable modelling also showed that PI‐RADS 4 and 5 remained strongly associated with increased odds of csPCa detection, independent of HoLEP status, reinforcing its prognostic validity across both populations.

Our findings differ from those of Pellegrino et al.,^14^ who reported compromised mpMRI accuracy following prior prostate surgery, including HoLEP. Their study reported on a cohort of 24 patients with prior prostate surgery, pooling different procedures such as TURP and HoLEP. The specific number of HoLEP patients was not reported, and the small overall sample size limits conclusions regarding any individual surgical technique. In contrast, our cohort of 97 HoLEP patients, though still modest, represents the largest to date focused solely on HoLEP and demonstrates that PI‐RADS retains diagnostic value post‐procedure. Given that HoLEP uses a holmium laser with limited tissue penetrance, the procedure may result in less inflammation and scarring compared to TURP, which could account for its more favourable impact on subsequent mpMRI interpretation.^16^, ^17^ This distinction may help explain the preserved diagnostic utility of PI‐RADS in our study.

On multivariable logistic regression, prior HoLEP was not independently associated with lower odds of csPCa detection after adjusting for clinical and imaging variables. This further supports the notion that PI‐RADS scored mpMRI remains reliable post‐HoLEP. Our interaction model did not demonstrate significant effect modification by HoLEP status, although the analysis was underpowered and should be interpreted cautiously.

This study is not without its limitations. The small size of the HoLEP cohort limits statistical power and may preclude detection of subtle differences in diagnostic accuracy. Additionally, retrospective design and lack of centralized radiologic review introduce potential for classification bias. Nonetheless, the use of a large, contemporary, real‐world dataset and consistent application of PI‐RADS v2.1 criteria strengthens the generalizability of our findings.

These findings have important clinical implications, considering mpMRI has become a cornerstone in prostate cancer diagnosis, biopsy guidance and risk stratification and that HoLEP is an increasingly utilized prostate outlet procedure. Urologists and radiologists may hesitate to rely on mpMRI in patients with altered anatomy following HoLEP, fearing decreased diagnostic accuracy or misleading PI‐RADS scores.^17^, ^18^ Our data suggest that such concerns may be overstated and that PI‐RADS retains its clinical utility even in this setting. This supports continued use of mpMRI to guide biopsy decisions in patients with prior HoLEP, potentially reducing unnecessary biopsies while preserving sensitivity for csPCa.

Future studies should aim to validate these findings in larger, multicentre cohorts and further investigate the imaging characteristics of post‐HoLEP prostates that may mimic or mask cancer. Prospective studies could also incorporate diagnostic performance and interobserver variability to assess the reproducibility of PI‐RADS scoring post‐HoLEP.

## 5 CONCLUSION

In conclusion, prior HoLEP does not appear to significantly impair the diagnostic accuracy of mpMRI for detecting csPCa when interpreted using PI‐RADS. These findings support the use and reliability of PI‐RADS for mpMRI in patients who have previously undergone HoLEP and are being surveilled for prostate cancer.

## AUTHOR CONTRIBUTIONS

Austin Drysch, Kathryn E. Fink, Nicole Handa, Mitchell M. Huang, Ridwan Alam, Amy E. Krambeck, Hiten D. Patel and Ashley E. Ross contributed to the conception and design of the study. Austin Drysch, Kathryn E. Fink and Yutai Li acquired the data. Austin Drysch, Sai Kumar and Yutai Li analyzed and interpreted the data. Austin Drysch and Kathryn E. Fink drafted the manuscript, and all authors, including Nicole Handa, Mitchell M. Huang, Ridwan Alam, Amy E. Krambeck, Hiten D. Patel and Ashley E. Ross, critically revised it for important intellectual content. Sai Kumar performed the statistical analysis. Ridwan Alam, Amy E. Krambeck, Hiten D. Patel and Ashley E. Ross provided supervision. No authors reported obtaining funding or providing administrative, technical or material support. All authors approved the final version of the manuscript and agree to be accountable for all aspects of the work.

## CONFLICT OF INTEREST STATEMENT

Amy E. Krambeck is a consultant for Richard Wolf, Storz, and Boston Scientific Corporation. Hiten D. Patel is supported by the Department of Defense Prostate Cancer Research Program (HT9425‐25‐1‐0498), a Prostate Cancer Foundation Young Investigator Award (24YOUN22) and a Developmental Research Program grant from the Polsky Urologic Cancer Institute and SPORE in Prostate Cancer at the Robert H. Lurie Comprehensive Cancer Center (P50CA180995). Hiten D. Patel served on a one‐time Advisory Board for Cleveland Diagnostics and consultant for A3P Biomedical. Ashley E. Ross is a consultant for Astellas, Bayer, Blue Earth, Boston Scientific Corporation, BilliontoOne, Astra Zenneca, Janssen, Lantheus, Veracyte and Pfizer.

## References

[1] Cornford P , van den Bergh RCN , Briers E , van den Broeck T , Brunckhorst O , Darraugh J , et al. EAU‐EANM‐ESTRO‐ESUR‐ISUP‐SIOG Guidelines on Prostate Cancer‐2024 update. Part I: screening, diagnosis, and local treatment with curative intent. Eur Urol. 2024;86(2):148–163. 10.1016/j.eururo.2024.03.027 38614820

[2] Kasivisvanathan V , Rannikko AS , Borghi M , Panebianco V , Mynderse LA , Vaarala MH , et al. MRI‐targeted or standard biopsy for prostate‐cancer diagnosis. N Engl J Med. 2018;378(19):1767–1777. 10.1056/NEJMoa1801993 29552975 PMC9084630

[3] Hugosson J , Mansson M , Wallstrom J , Axcrona U , Carlsson SV , Egevad L , et al. Prostate cancer screening with PSA and MRI followed by targeted biopsy only. N Engl J Med. 2022;387(23):2126–2137. 10.1056/NEJMoa2209454 36477032 PMC9870590

[4] Rouviere O , Puech P , Renard‐Penna R , Claudon M , Roy C , Mège‐Lechevallier F , et al. Use of prostate systematic and targeted biopsy on the basis of multiparametric MRI in biopsy‐naive patients (MRI‐FIRST): a prospective, multicentre, paired diagnostic study. Lancet Oncol. 2019;20(1):100–109. 10.1016/S1470-2045(18)30569-2 30470502

[5] Mazzone E , Stabile A , Pellegrino F , Basile G , Cignoli D , Cirulli GO , et al. Positive predictive value of prostate imaging reporting and data system version 2 for the detection of clinically significant prostate cancer: a systematic review and meta‐analysis. Eur Urol Oncol. 2021;4(5):697–713. 10.1016/j.euo.2020.12.004 33358543

[6] Oerther B , Engel H , Bamberg F , Sigle A , Gratzke C , Benndorf M . Cancer detection rates of the PI‐RADSv2.1 assessment categories: systematic review and meta‐analysis on lesion level and patient level. Prostate Cancer Prostatic Dis. 2022;25(2):256–263. 10.1038/s41391-021-00417-1 34230616 PMC9184264

[7] Woo S , Suh CH , Kim SY , Cho JY , Kim SH . Diagnostic performance of prostate imaging reporting and data system version 2 for detection of prostate cancer: a systematic review and diagnostic meta‐analysis. Eur Urol. 2017;72(2):177–188. 10.1016/j.eururo.2017.01.042 28196723

[8] Sandhu JS , Bixler BR , Dahm P , Goueli R , Kirkby E , Stoffel JT , et al. Management of lower urinary tract symptoms attributed to benign prostatic hyperplasia (BPH): AUA Guideline Amendment 2023. J Urol. 2024;211(1):11–19. 10.1097/JU.0000000000003698 37706750

[9] Robles J , Shin YE , Rojanasarot S , Miller NL . Niche no more? Mapping US trends and regional disparities in holmium laser enucleation of the prostate from 2018 to 2022. J Endourol. 2025;39(8):781–787. 10.1089/end.2025.0188 40587330

[10] Sakai A , Borza T , Antar A , Richmond E , Allen GO , Knoedler M , et al. Incidental prostate cancer diagnosis is common after holmium laser enucleation of the prostate. Urology. 2024;183:170–175. 10.1016/j.urology.2023.11.014 38043905 PMC10872358

[11] Rosenkrantz AB , Taneja SS . Radiologist, be aware: ten pitfalls that confound the interpretation of multiparametric prostate MRI. AJR am J Roentgenol. 2014;202(1):109–120. 10.2214/AJR.13.10699 24370135

[12] Panebianco V , Giganti F , Kitzing YX , Cornud F , Campa R , de Rubeis G , et al. An update of pitfalls in prostate mpMRI: a practical approach through the lens of PI‐RADS v. 2 guidelines. Insights Imaging. 2018;9(1):87–101. 10.1007/s13244-017-0578-x 29063480 PMC5825307

[13] Quon JS , Moosavi B , Khanna M , Flood TA , Lim CS , Schieda N . False positive and false negative diagnoses of prostate cancer at multi‐parametric prostate MRI in active surveillance. Insights Imaging. 2015;6(4):449–463. 10.1007/s13244-015-0411-3 PMC4519810 26002487

[14] Pellegrino F , Stabile A , Mazzone E , Sorce G , Barletta F , de Angelis M , et al. Does previous prostate surgery affect multiparametric magnetic resonance imaging accuracy in detecting clinically significant prostate cancer? Results from a single institution series. Prostate. 2022;82(12):1170–1175. 10.1002/pros.24368 35538401

[15] Barentsz JO , Richenberg J , Clements R , Choyke P , Verma S , Villeirs G , et al. ESUR prostate MR guidelines 2012. Eur Radiol. 2012;22(4):746–757. 10.1007/s00330-011-2377-y 22322308 PMC3297750

[16] Gravas S , Bachmann A , Reich O , Roehrborn CG , Gilling PJ , de la Rosette J . Critical review of lasers in benign prostatic hyperplasia (BPH). BJU Int. 2011;107(7):1030–1043. 10.1111/j.1464-410X.2010.09954.x 21438974

[17] Han EA , Nandalur KR , Morgan MA , Arora SS , Loening AM , Bivalacqua TJ , et al. MRI of benign prostatic hyperplasia: important pre‐ and posttherapeutic considerations. Radiographics. 2023;43(5):e220096. 10.1148/rg.220096 37022958

[18] Diaz TA , Benson B , Clinkenbeard A , Long JR , Kawashima A , Yano M . MRI evaluation of patients before and after interventions for benign prostatic hyperplasia: an update. AJR am J Roentgenol. 2022;218(1):88–99. 10.2214/AJR.21.26278 34259037

